# Observations on Tic Douloureux

**Published:** 1814-05

**Authors:** 


*For the Medical and Physical Journal.*

*Observations on Tic Douloureux; by* Mr. MEGLIN.

ELIZ. WEEPE, aged thirty-four years, in the month of March 1812, was seized with acute pains in the right angle of the lower jaw, which extended over all the side of the face to the ala nasi, with fluttering sensations of the muscular parts. The symptoms returned in long and violent paroxysms after some hours of disturbed sleep, during which time there always remained a greater or less degree of dull pain. At the height of the paroxysm the pain extended over the occiput, and occupied the posterior half of the head to the crown. The complaint had existed in a most insupportable degree, several weeks previous to my seeing her. She had tried a variety of anti-rheumatic medicines without effect, and finally determined on the extraction of one or two of her teeth. For my advice in this respect I was consulted. I had no difficulty in pronouncing the disease to be the tic douloureux. My first care was to dissuade her from the extraction of the teeth, assuring her that I knew, from long experience, the operation would be unsuccessful; and that far from procuring herself relief from it, it would infallibly aggravate her complaint. I prescribed some pills composed of equal parts of extract of hyosciamus, valerian, and sublimed oxide of zinc. I desired her to take a pill containing three grains night and morning, and to increase the dose by one pill every time until she took ten; but cautioned her to diminish the quantity if they produced any particular uneasiness. She could not go beyond three at each dose. Four pills produced nausea, affection of the heart, fainting, vertigo, &c. These were continued three or four weeks, at which time she was manifestly relieved. In less than eight days they had entirely disappeared, and have not returned, though a period of five months has elapsed. It is a little remarkable that so small a dose of the remedy should have been efficacious in effecting so complete a cure.

Z. Y., aged 38, was affected with this complaint. It appeared to her to be common tooth-ache. The pain came on in violent paroxysms, principally about the last molares of the left upper jaw. As the patient had formerly suffered from carious teeth, she expected relief from their extraction, an operation she had usually submitted to. Eight different times it had been performed with success, the pain having immediately disappeared: she therefore naturally desired the removal of the suspected tooth, which was done. It was not diseased, and our patient for once was disappointed in the expectation expectation of obtaining relief. The pains continued, and became almost insupportable. Sometimes during the day spasms came on in the socket of the extracted tooth, and in the buccinator and masseter muscles, by paroxysms of greater or less duration, so violent, that the patient described it as though the parts were torn away with pinchers. To this was added so forcible a tetanic contraction of the lower jaw, that fluids could scarcely be introduced into the mouth. She has since declared to me, that in the excess of agony she was frequently tempted to put an end to her existence. A variety of remedies were employed previous to my being consulted. I recommended the same medicine as in the former case. Six pills were taken morning and night, and in three weeks the cure was complete. Four months have since elapsed without any relapse of the complaint.

This remedy has been also found serviceable in the case of the Abbé du C. though the effect has been less perfect than in the former instances. He had been affected with this complaint many years. M. du C. until the age of 40, had experienced rheumatic pains in the left knee. At this time they were exchanged for an acute pain in the right side of the face, coming on at intervals with great violence. It affected the right ala nasi, the cheek, the eye, and all the upper part of the head. This pain was extremely acute, and its accession very frequent and sudden. It was compared to an electric shock. Various treatment was adopted with little success. He occasionally had intervals of some months, but the pains returned again with the same force at the different changes of the seasons. Once he experienced an interval of eighteen months, but the return of the pain was marked by a great accession of violence, and which continued to increase in strength and durat on as his age advanced. Many anodyne remedies were administered, an issue was made in the leg, a seton put into the neck, and teeth were drawn, without effect. A copious salivation, especially in the morning, came on spontaneously. The medicine above-mentioned was recommended to him, which he took in the quantity of six pills (eighteen grains) night and morning, drinking after it Cyathum Infus. Folior. Tiliæ and Florum Aurantii. After some days he was evidently better. The pains diminished in intensity, and frequently the salivation ceased. The sensibility of the part lessened so as to enable him to bear pressure without pain. One effect perceived from the remedy was a great flow of frothy urine, and a dryness of the mouth, which was neither painful nor troublesome, and which remained half an hour after after taking it. He was now able to walk out, after having been eighteen months a prisoner, and resumed his usual occupations; but the variations of the weather, which are very frequent in this climate, occasion slight relapses. His stomach is not inconvenienced by the medicine, and his appetite is good. At first, while under the use of the medicine, he occasionally felt shocks of an electric kind in all parts of the body, but they have now ceased.—*Journale de Medecine, par Corvissart, Leroux, & Boyer.*

